# The intersection of oncology and oral health: exploring nurses’ insights and practices — a systematic review

**DOI:** 10.1007/s00520-024-08317-5

**Published:** 2024-01-30

**Authors:** Arsheen Imran Sajwani, Mohammad AlShdaifat, Fatima Hashi, Eman Abdelghany, Ibrahim Alananzeh

**Affiliations:** 1 School of Nursing, University of Wollongong Dubai, Dubai, United Arab Emirates; 2 Waist Health Center, Primary Health Care Centers, Sharjah Health Services, Sharjah, United Arab Emirates; 3 School of Nursing Faculty of Science, Medicine & Health, University of Wollongong, Dubai, United Arab Emirates

**Keywords:** Cancer patient, Chemotherapy, Knowledge, Nurses, Oral health management, Oral care, Patient quality of life, Tumor patient

## Abstract

**Purpose:**

Oral health care for cancer patients is essential but often overlooked. Nurses play a critical role in assessing and managing oral health in this population. This systematic review aims to examine nurses’ knowledge, attitudes, and practices regarding oral healthcare in cancer patients.

**Methods:**

A systematic review was conducted following the Joanna Briggs Institute methodology. Qualitative and quantitative studies focused on nurses’ knowledge, attitudes, and practices in oral healthcare for cancer patients. Seven databases were searched for studies published between January 2000 and January 2023. The primary outcomes of interest were patient satisfaction, quality of life, and nurses’ knowledge, attitudes, and practices related to oral healthcare.

**Results:**

The review identified gaps in nurses’ knowledge and training in oral healthcare for cancer patients. Insufficient understanding of oral diagnoses, treatment protocols, and pediatric oral care was noted. Lack of knowledge and skills posed barriers to implementation. Some healthcare providers demonstrated low awareness of oral health recommendations, including the use of fluoridated toothpaste and the need for dental referrals. Referrals to dental services and regular oral assessments were infrequent. Attitudes towards oral healthcare varied, with providers feeling more comfortable in certain areas than others.

**Conclusion:**

Enhancing nurses’ knowledge, attitudes, and practices in oral healthcare for cancer patients is crucial. Targeted educational initiatives and interventions are needed to address these gaps. By improving nurses’ understanding of oral complications and management approaches, patient outcomes and quality of life can be improved.

**Registration**: PROSPERO International prospective register of systematic reviews, ID: CRD42022368053.

**Supplementary Information:**

The online version contains supplementary material available at 10.1007/s00520-024-08317-5.

## Background

In recent years, oral health care has been recognized as a vital aspect of maintaining overall well-being [1, 6, 11, 13]. However, other patients’ medical concerns overshadow oral assessment and management, especially cancer patients [6, 13]. Consequently, oral health concerns will initiate other health risks and potential complications if neglected and untreated [3, 6]. Nurses play a vital role in patient care management as frontline care providers in assessing, managing, and promoting oral health for cancer patients undergoing treatment.

Cancer therapy has many side effects related to radiation, and chemotherapy can significantly impact patients’ oral health, treatment outcomes, and overall quality of life [2, 5]. These adverse effects can be oral mucositis [7, 12] (inflammation and ulceration in the inner mucous membranes of the mouth) [2, 5, 13], xerostomia or dry mouth (related to less productivity of saliva) [5, 12], taste alteration [5, 10], and gingivitis and weakness of the immune system causing viral or fungal infections as thrush and cold sores [10]. As a result of these adverse effects, there is a significant impact on the patient’s health [12], such as a feeling of oral discomfort, pain [5], dental caries [5] or tooth loss [10], oral infections [7], speaking difficulty, gum inflammation and bleeding [7], appetite alteration and difficulty of eating and swallowing leads to poor nutritional intake [5, 7, 13], dehydration, as well as negative emotions [2, 5]. One published study in the Journal of Supportive Oncology reported that around 40–80% of cancer patients undertaking chemotherapy complained of oral mucositis as a side effect [2]. In addition, approximately 70–90% of cancer patients undergoing radiation therapy for the neck and head complained of acute oral mucositis [2]. Therefore, the role of health care providers in oral assessing [13] and reporting any patient’s oral health concern is crucial to enhance the treatment process and minimize the impacts of these side effects [6, 9].

Understanding the advantages of improving nursing performance towards oral healthcare for cancer patients involves acquiring adequate knowledge and proper awareness of identifying early signs [5, 6], implementing preventive measures [6], delivering oral hygiene instructions, communicating with other healthcare team members [2], educating patients [4], and intervening using effective strategies [6, 13]. Providing support and empathy helps in improving patients’ outcomes [13]. However, some studies pointed to potential gaps and a lack of sufficient nurses’ knowledge and training regarding cancer patients’ oral health management, leading to improper oral care practices causing suboptimal outcomes [6, 10]. A study reported that numerous nurses had insufficient knowledge about oral healthcare, especially the relationship between oral health and general well-being [1, 11]. The study recommended upgrading education and training programs to enrich nurses’ knowledge and improve oral healthcare practices [1, 10, 11].

On the other hand, some studies refer to educational interventions and training programs to effectively improve nurses’ knowledge and attitudes towards oral healthcare [9, 13]. Therefore, updated knowledge [12] empowers nurses to acquire positive attitudes and use the latest evidence-based practices [8] to provide holistic care, evaluating current practices for gaps and areas that need improvement, which can be achieved by delivering standardized protocols [4, 14], continuous education, and integrated oral care training programs [11, 12]. A study of supportive cancer care highlighted the benefits of an integrated oral care program for cancer cases. This program emphasizes the collaboration of nurses, oncologists, dentists, and other healthcare professionals [9]. Nurses’ active involvement can significantly promote treatment tolerance, optimize the best possible outcomes, and enhance oral health-related quality of life [8, 12].

This study aims to examine the current knowledge, attitudes, and nursing practices toward oral healthcare for cancer patients. Measuring their awareness and understanding of potential oral complications and management methods can visibly identify gaps in knowledge and training. In addition, exploring nurses’ attitudes towards oral care and their perceived barriers to implementing integrated oral care programs will assist in the progress of targeted educational initiatives and intervention strategies.

Ultimately, this review endeavours to participate in the body of knowledge associated with nurses’ role in oral healthcare for cancer patients. As a result of improving nurses’ knowledge, attitudes, and practices in this critical area of cancer care, we can struggle for enhanced patient outcomes, improved quality of life, and better overall healthcare delivery for patients undergoing cancer treatment.

## Review question

What are the nurse’s knowledge, attitudes, and practices towards oral healthcare? What are the potential benefits of the integrated oral care program on the people affected by cancer quality of life?

## Methods

### Search strategy

A comprehensive search strategy was developed to identify relevant studies for this systematic review. The search strategy included both qualitative and quantitative studies focusing on nurses’ knowledge, attitudes, and practices towards oral health care for cancer patients. The primary outcomes of interest were patient satisfaction and quality of life. The search was conducted in seven databases: CINAHL, Cochrane, Medline, PubMed, ScienceDirect, Scopus, and Google Scholar. The search was limited to peer-reviewed publications published in English between January 2000 and January 2023. The search strategy utilized a combination of keywords and subject headings related to oral health management, patient quality of life, nurses’ knowledge, cancer patients, and chemotherapy. The following keywords and their variations were used: “Oral health management,” “Oral Health,” “patient quality of life,” “Nurses’ knowledge,” “Cancer patient,” and “Chemotherapy.”

To ensure the search was comprehensive, the search strategy was reviewed and refined by two researchers. To minimize the risk of publication bias, reference lists of the included studies were also hand-searched for additional relevant articles. Furthermore, a PROSPERO search was performed to ensure that no similar systematic reviews had been conducted previously. Table 1 provides a detailed overview of the search terms used and the databases searched.

**Table 1:** Search strategy

Database 2000–2023	Search terms	Papers retrieved
CINAHL	"Cancer patient" OR "tumor patient" AND "chemotherapy" AND "knowledge" AND "nurses" AND "oral health management" OR "oral Care" AND "patient quality of life"	433
Cochrane	"Cancer patient" OR "tumor patient" AND "chemotherapy" AND "knowledge" AND "nurses" AND "oral health management" OR "oral Care" AND "patient quality of life"	6
Google Scholar	"Cancer patient" OR "tumor patient" AND "chemotherapy" AND "knowledge" AND "nurses" AND "oral health management" OR "oral Care" AND "patient quality of life"	31
Medline	"Cancer patient" OR "tumor patient" AND "chemotherapy" AND "knowledge" AND "nurses" AND "oral health management" OR "oral Care" AND "patient quality of life"	190
PubMed	"Cancer patient" OR "tumor patient" AND "chemotherapy" AND "knowledge" AND "nurses" AND "oral health management" OR "oral Care" AND "patient quality of life"	2472
ScienceDirect	"Cancer patient" OR "tumor patient" AND "chemotherapy" AND "knowledge" AND "nurses" AND "oral health management" OR "oral Care" AND "patient quality of life"	53
Scopus	"Cancer patient" OR "tumor patient" AND "chemotherapy" AND "knowledge" AND "nurses" AND "oral health management" OR "oral Care" AND "patient quality of life"	183

### Inclusion and exclusion criteria

The inclusion criteria for this systematic review encompass various types of research studies, both qualitative and quantitative, such as cross-sectional, cohort, case–control, randomized controlled trials (RCTs), and observational studies. These selected studies must have a specific focus on examining nurses’ knowledge, attitudes, and practices related to oral healthcare for patients with cancer across all age groups (adult and pediatric cancer survivors). Additionally, the review encompasses studies that investigate the potential benefits of integrated oral care programs on the quality of life of individuals affected by cancer. The primary outcomes of interest include patient satisfaction with the oral healthcare provided by nurses and the quality of life of cancer patients.

Conversely, the exclusion criteria aim to filter out studies that do not meet the inclusion criteria. This involves excluding studies that do not explore the potential benefits of integrated oral care programs on the quality of life of individuals affected by cancer. Non-peer-reviewed materials are also excluded. Studies conducted on populations other than nurses and cancer patients are not considered. Furthermore, studies lacking relevant outcome measures related to patient satisfaction and quality of life are excluded, as are those with insufficient data, incomplete reporting, or redundant content. These criteria ensure a focused and high-quality selection of studies that align with the research objectives.

### Study selection

**Fig. 1:**
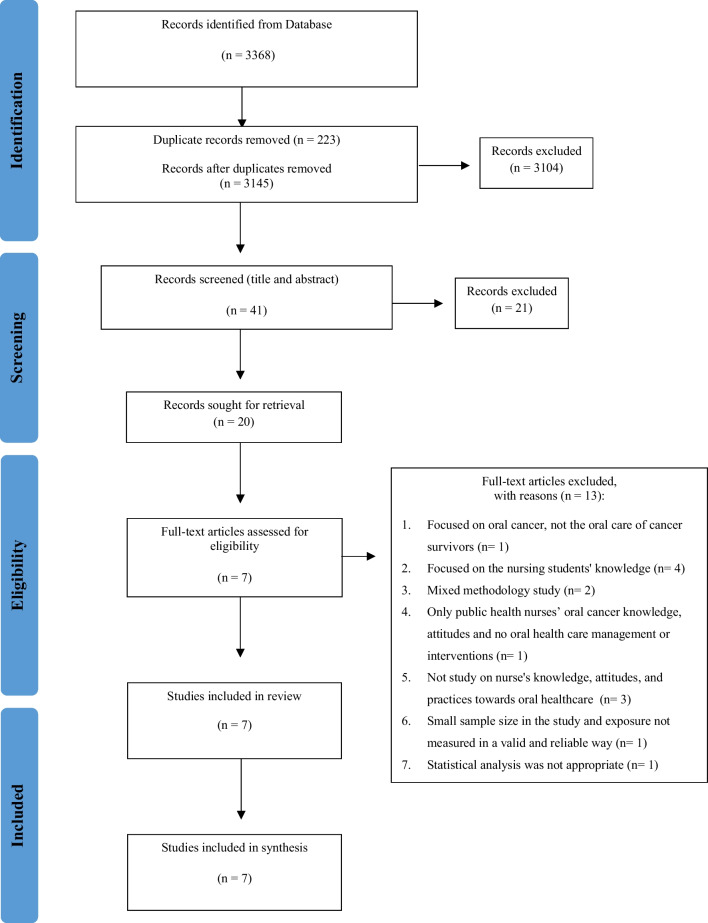
PRISMA flow diagram of the systematic review process

All studies identified from all databases were uploaded to Endnote X7 (Clarivate Analytics). Title and abstract were screened by two reviewers (MA and FH) as an initial step to determine study eligibility and to remove duplicates. The full texts of these potentially eligible studies were retrieved and imported into the JBI System for Unified Management, Assessment, and Review of Information (JBI SUMARI) [15]. (Piper, 2019). Full-text screening to assess inclusion and exclusion criteria was completed by three reviewers (MA, FH, and AS). Any disagreements or doubts regarding the eligibility of a specific study were clarified and resolved by an expert (full list of excluded study presented in Appendix A). The reference lists of all the inclusive studies were checked and verified for potential relative additional studies. Selection process presented in the PRISMA flow diagram (Fig. 1).

### Quality assessment

Two independent reviewers (AS and MA) appraised each study’s methodological quality using the JBI critical appraisal tool, with a third reviewer (IA or FH) conducting a check. Each criterion within the tool was assigned a score: yes = 2, no = 0, and unclear = 1. These scores were then converted into percentages. Based on the methodological quality assessment, no studies were excluded as all of them scored a minimum of 70% (Table 2). The JBI Grades of Recommendation were applied as follows: “grade A” signifies a “strong” recommendation for a particular health management strategy when specific conditions are met, including clear evidence that the desirable effects outweigh the undesirable effects, the presence of high-quality supporting evidence, no adverse impact on resource use, and consideration of values, preferences, and patient experience. Conversely, “grade B” indicates a weaker recommendation compared to “grade A.”

**Table 2:** Critical appraisal results

Cross-sectional studies																																
Citation					Q1				Q2			Q3			Q4				Q5			Q6			Q7			Q8				%
Tewogbade A, FitzGerald K, Prachyl D, Zurn D, Wilson C. 2008					Y				Y			Y			Y				Y			Y			N			N/A				75.0
Wei X, Jing M, Zhang X, Li C, Li L. 2022					Y				Y			Y			Y				N/A			N/A			Y			Y				75.0
Perry AD, Hiroko I, Patton LL, Wilder RS. 2015					N				Y			Y			Y				N/A			N/A			Y			Y				75.0
Suminski JA, Inglehart M, Munz SM, Van Poznak CH, Taichman LS. 2017					Y				Y			Y			Y				UC			UC			Y			Y				75.0
Southern H. 2007					Y				Y			Y			Y				Y			UC			Y			Y				87.5
%					80.0				100.0			100.0			100.0				40.0			20.0			80.0			80.0				
Randomized controlled trials																																
Citation	Q1	Q2	Q3	Q4		Q5		Q6			Q7			Q8			Q9			Q10			Q11			Q12				Q13		%
Pai R, Ongole R, Banerjee S, Prasad K, George L, George A, et al. 2019	Y	Y	Y	N		N		Y			Y			Y			Y			Y			Y			Y				Y		84.6
%	100	100	100	0		0		100			100			100			100			100			100			100				100		
Quasi-experimental studies (non-randomized experimental studies)																																
Citation							Q1			Q2			Q3			Q4		Q5			Q6			Q7			Q8		Q9		%	
Wårdh I, Paulsson G, Fridlund B. 2009							Y			Y			Y			N		Y			N/A			Y			Y		Y		77.7	
%							100			100			100			0		100			0			100			100		100			

Cross-sectional studies: (1) Were the criteria for inclusion in the sample clearly defined? (2) Were the study subjects and the setting described in detail? (3) Was the exposure measured in a valid and reliable way? (4) Were objective, standard criteria used for measurement of the condition? (5) Were confounding factors identified? (6) Were strategies to deal with confounding factors stated? (7) Were the outcomes measured in a valid and reliable way? (8) Was appropriate statistical analysis used? Randomized controlled trials: (1) Was true randomization used for assignment of participants to treatment groups? (2) Was allocation to groups concealed? (3) Were treatment groups similar at the baseline? (4) Were participants blind to treatment assignment? (5) Were those delivering treatment blind to treatment assignment? (6) Were outcomes assessors blind to treatment assignment? (7) Were treatment groups treated identically other than the intervention of interest? (8) Was follow-up complete, and if not, were differences between groups in terms of their follow-up adequately described and analyzed? (9) Were participants analyzed in the groups to which they were randomized? (10) Were outcomes measured in the same way for treatment groups? (11) Were outcomes measured in a reliable way? (12) Was appropriate statistical analysis used? (13) Was the trial design appropriate for the topic, and any deviations from the standard RCT design accounted for in the conduct and analysis? Quasi-experimental studies (non-randomized experimental studies): (1) Is it clear in the study what is the “cause” and what is the “effect” (i.e., there is no confusion about which variable comes first)? (2) Were the participants included in any comparisons similar? (3) Were the participants included in any comparisons receiving similar treatment/care, other than the exposure or intervention of interest? (4) Was there a control group? (5) Were there multiple measurements of the outcome both pre and post the intervention/exposure? (6) Was follow up complete and if not, were differences between groups in terms of their follow up adequately described and analyzed? (7) Were the outcomes of participants included in any comparisons measured in the same way? (8) Were outcomes measured in a reliable way? (9) Was appropriate statistical analysis used?

*Y* yes, *N* no, *UC* unclear, *N/A* not applicable

### Data extraction

The data extraction process involved the use of the JBI extraction tool [15]. Two review authors (IA, AS) independently and in duplicate extracted the required data from the included studies. In cases of disagreements, a third review author (FH) provided adjudication to resolve any discrepancies. The extracted data encompassed various aspects, including general study information such as title, authors, contact address, publication source, and publication year. Additionally, data pertaining to nurses’ knowledge, practice, and attitude towards managing oral healthcare were gathered and are presented in Table 3.

**Table 3:** Characteristics of included studies

Study	Country	Setting/context	Participant characteristics	Groups	Outcomes measured	Main description of results
Analytical cross-sectional study form						
Tewogbade A, FitzGerald K, Prachyl D, Zurn D, Wilson C. 2008	USA	This study was carried out in the Center for Cancer and Blood Disorders (CCBD) Unit of Children’s Medical Center, Dallas, Texas (CMC)	Pediatric oncology nurses **Questionnaires were completed by 33 pediatric oncology nurses:** 12 had been licensed for 1 to 3 years, and 11 had been licensed for more than 6 years **Experiences:** Three of the nurses had been working on the unit for less than 1 year, 13 had been working on the unit for 1 to 3 years, 7 had been working on the unit between 3 and 6 years, and 10 had been there more than 6 years	33 pediatric oncology nurses	Nurse’s understanding of oral health for hematology and oncology patients	The study examined nurses’ knowledge and practice for different categories: 1) Oral examination: variation among nurses in conducting oral cavity evaluation for both patients with cancer and those undergoing hematopoietic stem call treatment (HSCT) 2) Dental referrals: 3/4 of the participants did not make dental referral; for patients after chemotherapy 3) Oral hygiene instruction (OHI): majority of nurses distributed oral hygiene aid and instructed parents to use them 4) Diagnosis and treatment: Nurses vary in their knowledge of oral diagnosis, treatment, disease etiologies, and hygiene protocols; however, the majority are able to give proper differential diagnoses of mucositis but were more knowledgeable about the proper treatment of fungal infection. Also, the nurses had more difficulty with the diagnosis and treatment of xerostomia An oral care intervention was developed based on the above finding; the new protocol contains 6 steps: 1. Gather information—criteria to determine patient risk and referral 2. Oral hygiene protocol—specific instructions on brushing and flossing techniques were given 3. Viral infection—instruction on treating an oral viral infection 4. Fungal infection—instruction on treating an oral fungal infection 5. Oral assessment guide—allow nurses to adequately diagnose conditions that they were not previously able to recognize 6. Oral care algorithm—an oral care algorithm The following are the summary of finding: Nurse’s training and beliefs about oral care would make more difference in the patient oral hygiene instruction than nurses experience Examination of patients undergoing chemotherapy and/or radiotherapy appeared to be conducted more frequently than for patients undergoing HSCT Referrals to dental services are possibly the most important avenue for children to receive oral care while undergoing treatment for cancer or prior to HSCT To sum up, the nurses surveyed have less than an adequate knowledge of how to provide oral care for children undergoing cancer treatment and HSCT
Wei X, Jing M, Zhang X, Li C, Li L. 2022	China	19 ICUs of 11 tertiary hospitals from Henan province in China	173 nurses and 19 head nurses online using a structured questionnaire	173 nurses and 19 head nurses. All of the participants were registered nurses and had worked in the ICU. Also, they have conducted oral care for postoperative patients with oral cancer at least 6 times in the past month	What are the practicing situation of nurses in the intensive care unit (ICU) for postoperative patients with oral cancer and their need for training	The majority of participants claimed that the lack of knowledge and skills surrounding oral care was the main barrier for nurses to implement oral care ICU nurses had no continuing education or training in oral care for postoperative patients with oral cancer Participants stated their preference to receive training on oral care specifically about the indication, contraindication, tools, and mouthwash. On-hand training (scenario simulation) was the preferred method of training. To sum up, the finding shows diversity in practice among groups, lack of oral care knowledge, and the pertinent need for education. Therefore, a standard protocol or clinical practice guidelines for oral care for postoperative patients with oral cancer should be developed to equip nurses with the required skills to deliver quality oral care
Perry AD, Hiroko I, Patton LL, Wilder RS. 2015	USA	The study was carried out in the Association of Pediatric Hematology and Oncology Nurses’ (APHON) 36th Annual Conference and Exhibit on October 4 to 6, 2012, in Pittsburgh, Pennsylvania	Pediatric oncology, pediatric oncology, or hematology nurse: 97% were women 70% work 36 h or more a week 54% work as certified pediatric oncology/hematology nurses 53% been employed as a pediatric oncology nurse for 10 or more years 60% did not have a clinical requirement regarding the assessment of the teeth and gums during their nursing school education	235 pediatric oncology, pediatric oncology, or hematology nurse	Pediatric oncology and hematology nurses’ (1) knowledge, (2) perceived ability, and (3) practice behaviors in assisting with the various oral health care needs of pediatric oncology patients (4) Their demographic characteristics and oral health competencies	**Knowledge**: 100% were aware of potential oral complications related to cancer treatment Awareness level related to the professional oral health care recommendations for pediatric oncology patients: The use of a soft bristled toothbrush (97%) Daily inspection to determine the presence or absence of oral complications (87%) The use of fluoridated toothpaste 57% Referrals to a dentist for consultation prior to cancer treatment 29% Overall, only 14% of survey participants responded correctly to all informative questions that assessed their knowledge of oral health care recommendations for pediatric oncology patients undergoing cancer treatment **Perceived ability:** • 77% reported that they are comfortable performing oral procedures • 72% are adequately trained to provide oral health care instructions/education • 84% adequately trained to perform oral care procedures • 70% were very confident in examining for the presence of oral pain and providing oral hygiene instructions • Less than half were very confident in their ability to examine the health of teeth and gums for complications of trismus, dysphagia, and xerostomia **Practice behaviors**: • 60% reported examining all of their patients for the presence of oral pathology or oral pain • Half of the participants examine all of their pediatric oncology patients’ teeth and/or gums, detect dysphasia, and provide instructions • 40% or less reported examining all patients for the presence of xerostomia, trismus, and discussing the importance of seeking routine professional dental care • More than one-third reported referring patients to dental professionals prior to the initiation of cancer treatment (39%) and/or during cancer treatment (31%) • 20% reported never referring patients to dental professionals **Oncology nurses’ demographic characteristics and oral health competencies** • Survey respondents who had a clinical requirement regarding oral health assessment during nursing education presented greater oral health related knowledge and confidence in addition to providing oral care instructions and examining the patient’s mouth more often • More nurses who work full time in direct patient care and have a source for dental referrals responded to oral health knowledge questions correctly • Job title or being certified as oncology nurse and hours spent in oral health education/training during nursing school were not associated with oral health competencies
Suminski JA, Inglehart M, Munz SM, Van Poznak CH, Taichman LS. 2017	Ann Arbor	University of Michigan	**Included:** – 194 responses from 5000 emails – (*n* = 164) meeting study eligibility **Excluded:** 29 respondents	Registered oncology nurses or nurse practitioners with a background in treating patients with breast cancer	• Education competency of staff to provide oral instructions • Educating patients about oral health during their visits • Percentage of staff who are performing oral assessment with and without dental specialist collaboration	164 participants: *The majority often or very often educated their patients about oral health (*n* = 69, 42%) *Staff well educated to provide oral health instructions report was as follows: - Only 51 (31%) were well or very well educated - 58 (35%) had sufficient knowledge and confidence to perform oral health assessments *For staff performing oral assessments: - Only 56 (34%) used a tongue depressor or flashlight - 28 (17%) performed patient oral hygiene care - Only 20 (12%) often or very often referred their patients to a dental specialist - 16 (10%) collaborated with dental specialists during their patients’ treatment *Barriers: - Only 37 (23%) considered a lack of time as a barrier
Southern H. 2007	Ireland	One urban health care institution over a 3-month time frame	**Included:** *Total sample (*n* = 100): 72 completed the questionnaire – 37 (51.4%) were general nurses – 35 (48.6%) were oncology nurses	Registered general nurses/specialist cancer nurses	• **Knowledge and education of oral care and oral health assessment** • **Management of oral care:** - Information on oral complications - Examination of the oral cavity - Documentation of oral cavity complications or changes - Oral care routines - Influences on knowledge of oral care and performed oral care according to nurses’ age and how often oral hygiene information was provided to patients	• Study *p*-value of < 0.05 • Nurses lack adequate knowledge of oral care and oral health assessment • Continuing education in oral care for nurses is minimal • There is a lack of dentistry involvement in nurse education and nursing practice in relation to oral care *Knowledge and education of oral care and oral health assessment: **General nurse education:** - 33 (45.8%) respondents received both theoretical and clinical education in oral care - (*n* = 7, 9.7%) received lot of education - (*n* = 31, 43.1%) received no education on cytotoxic drugs or radiation treatment **Oncology nurse education:** - 22 (62.9%) had received both - Need for continuous education was reported by (*n* = 68, 94.5%) of all oncology nurses *Management of oral care: - (*n* = 47, 65.3%) staff were informing patients about oral complications - (*n* = 59, 82.1%) are recommended daily examination of a patient’s oral cavity, only (*n* = 47, 65.1%) are performing daily and (*n* = 12, 16.6%) more often than daily - (*n* = 34,91.9%) of general nurses and (*n* = 30, 88.2%) are always documenting of oral cavity findings - (*n* = 49, 68.1%) of cancer patients reported of performing oral care - There was a statistically significant main effect for age [*F* (2.63) = 3.302, *p* = 0.043] nurses who were significantly younger and had greater total self-rated knowledge scores are giving education about oral care for patients - (*n* = 41, 56.9%) reported feeling comfortable in examining a patient’s oral cavity and nurses who reported dissatisfied were showing less knowledge about oral abnormalities symptoms - (*n* = 57, 79.2%) reported that patients are receiving referral to hospital dentist. (*n* = 21, 29.2%) received great support, (*n* = 20, 27.8%) received some support, (*n* = 30, 41.6%) large number never received sufficient dental support
Randomized controlled trial						
Pai R, Ongole R, Banerjee S, Prasad K, George L, George A, et al. 2019	India	This study is a randomized, outcome assessor blinded study conducted in a tertiary care hospital in South India in 2 phases. For phase I, the staff nurses were trained on oral care in cancer patients, and for phase II, randomized clinical trial was used to determine the effectiveness of oral care protocol	**Patients’ characteristics (70):** Patients planned for radiation to the head and neck region, patients who are in any stage of cancer receiving chemoradiation, only radiation or postoperative radiation • Patients in 18–75 years age group • At least 75% of both parotids are within the radiation field **Nurses’ characteristics (25):** staff nurses working in radiation oncology areas hospital	**Experimental arm: (intervention) oral care protocol** Patient in this arm receive oral care according to protocol which includes nursing assessment with oral health assessment tool and oral care kit **Control: (comparator agent) standard of care (SOC) of oral care** Patients in this control arm receive oral care according to SOC of oral care as per the hospital practices	**The primary outcome** is the Effectiveness of an oral care protocol on chemotherapy- and radiation therapy–induced oral complications in cancer patients by measuring: 1- The incidence of oral complications that will be collected from patient records 2- Oral health assessment tool **The secondary outcome measures** are the cost analysis, documentation audit, knowledge, and practice of staff nurses	The study is not yet completed. The results of the preliminary survey conducted among 158 staff nurses showed that 81 (51.3%) of the staff nurses had poor knowledge regarding oral care of cancer patients, and majority (128 (81.0%)) of them were suggested for training in the specific area of oral care of cancer patients. A pilot study conducted by the principal investigator to determine the feasibility of the study among 9 participants (4 experimental and 5 control) revealed that there was slight difference found in the incidence of oral complications among the group in relation to weeks of assessment • Swallowing difficulty, oral mucositis, infection, and the nutritional compromise were delayed in the experimental group Taste alteration, xerostomia, and bleeding gums appeared early in the experimental group in comparison with the control group
Quasi-experimental study						
Wårdh I, Paulsson G, Fridlund B. 2009	Sweden	This study, which had a pre-and post-non-randomized, quasi-experimental design with both intra- and inter-individual comparisons, was conducted on five wards at five different hospitals in Sweden	Registered nurses, *n* = 133 and auxiliary nurses, *n* = 109 on five wards at different hospitals providing cancer care. The nursing staff had similar composition according to education, age, and experience	Registered nurses, *n* = 133 and auxiliary nurses, *n* = 109	Long-lasting changes in the nursing staff’*s* understanding of oral health care for cancer patients after an oral health care intervention. For both registered nurses and axillary nurses	**Attitudes to oral health care:** No statistically significant improvements were demonstrated in terms of attitudes to oral health care after the intervention compared with the situation before the intervention **Implementation opportunities:** • Sufficient knowledge for the implementation of oral health care; significant changes could be seen both for registered nurses (*p* = 0.002) and for auxiliary nurses (*p* = 0.001) when comparisons were made before and after the intervention • Availability of aids/devices for assistance; there was an equal improvement for both registered nurses and auxiliary nurses (*p* = 0.002) • Familiarity with practical oral healthcare procedures improved for registered nurses (*p* = 0.04), but not for auxiliary nurses • The ability to give oral healthcare advice improved for both registered nurses (*p* = 0.01) and auxiliary nurses (*p* = 0.03). No changes could be seen in terms of time and ability to influence care receivers, reluctant to oral healthcare assistance **Knowledge of importance:** Both the registered nurses (*p* = 0.03) and the auxiliary nurses (*p* = 0.009) had less knowledge of importance of the diseases affecting the oral cavity after the intervention than before the intervention. The other aspects of knowledge of importance were not influenced by the intervention, in the case of either the registered nurses or the auxiliary nurses

### Data synthesis

The heterogeneity test was conducted to assess the variability among the included studies, and it revealed a high degree of heterogeneity in the studies measurement and outcomes, making it impossible to perform a meaningful meta-analysis. Therefore, alternative methods of synthesis and summary approaches were employed. The findings were presented in a narrative form to effectively convey the results to readers. It should be noted that due to the substantial heterogeneity observed, caution should be exercised when interpreting the overall findings.

## Results

### Characteristics of studies

The included studies were conducted across diverse geographical locations, spanning the USA, China, India, Sweden, and Ireland. This global distribution underscores the universal significance of addressing oral care practices in healthcare settings. The primary subjects of investigation in these studies were healthcare professionals, predominantly nurses, who played pivotal roles in the care of various patient populations. These encompassed pediatric oncology patients [16, 18], postoperative patients dealing with oral cancer [10, 17], and individuals undergoing radiation therapy. Several of the studies honed in on specific patient demographics. For instance, one study delved into oral care for pediatric oncology patients, shedding light on the distinctive challenges and considerations inherent in caring for children with cancer [16]. In contrast, another study specifically targeted postoperative patients with oral cancer, focusing on their unique needs and concerns within the realm of oral healthcare [17].

### Knowledge

According to study [16], nurses vary in their knowledge of oral diagnosis, treatment, disease etiologies, and hygiene protocols; however, the majority are able to give proper differential diagnoses of mucositis but were more knowledgeable about the proper treatment of fungal infection. Also, the nurses had more difficulty with the diagnosis and treatment of xerostomia. The nurses surveyed have less than an adequate knowledge of how to provide oral care for children undergoing cancer treatment and hematopoietic stem cell transplantation (HSCT).

The majority of participants in study [17] claimed that the lack of knowledge and skills surrounding oral care was the main barrier for nurses to implement oral care. ICU nurses had no continuing education or training in oral care for postoperative patients with oral cancer. Participants stated their preference to receive training on oral care specifically about the indication, contraindication, tools, and mouthwash. On-hand training (scenario simulation) was the preferred method of training.

A study [18] was conducted where 100% of the participants were aware of the potential oral complications related to cancer treatment. The study also revealed the level of awareness related to the professional oral health care recommendations for pediatric oncology patients, with 97% of the participants being aware of the use of a soft bristled toothbrush and 87% of the participants knowing the importance of daily inspection to determine the presence or absence of oral complications. Only 57% of the participants were aware of the use of fluoridated toothpaste, and 29% knew about the need for referrals to a dentist for consultation prior to cancer treatment. Overall, the study found that only 14% of the participants responded correctly to all informative questions that assessed their knowledge of oral health care recommendations for pediatric oncology patients undergoing cancer treatment.

According to a study [9] conducted, only 31% of staff members were considered well or very well educated to provide oral health instructions. About 35% had enough knowledge and confidence to perform oral health assessments, and the study [9, 19] showed a significant *p*-value of < 0.05. The study [19] also found that nurses lacked adequate knowledge of oral care and oral health assessment, and there was minimal continuing education in oral care for nurses. Additionally, there was a lack of dentistry involvement in nurse education and nursing practice regarding oral care. Among general nurse education, 45.8% of respondents received both theoretical and clinical education in oral care, while 43.1% received no education on cytotoxic drugs or radiation treatment. For oncology nurse education, 62.9% had received both, and 94.5% reported a need for continuous education. The study also revealed a statistically significant main effect for age, where younger nurses with higher total self-rated knowledge scores were more likely to provide education about oral care for patients. The study [2] reported that staff nurses (51.3%) had inadequate knowledge related to oral care of cancer patients, and majority (81.0%) of the nurses were recommended for training on the oral care of cancer patients.

### Practices

Four studies highlighted oral healthcare practices for oncology patients undergoing cancer treatment or HSCT. Referral to dental services was found to be a crucial avenue for children to receive oral care during cancer treatment or prior to HSCT; however, only a minority of healthcare providers made such referrals. Study [18] reported that while 60% of healthcare providers examined all their patients for the presence of oral pathology or oral pain, less than half examined patients for the presence of xerostomia and trismus. Moreover, only 39% reported referring patients to dental professionals before cancer treatment initiation, and 31% referred during treatment. A study [9] reported that only 12% of healthcare providers often or very often referred their patients to dental specialists, with time being the most commonly cited barrier to oral healthcare practices. The study [20] found no significant improvements in attitudes towards oral healthcare following an intervention among healthcare providers but reported improved familiarity with practical oral healthcare procedures and ability to give oral healthcare advice. In the study [2], it was identified that 34.2% of nurses did not perform oral care as a daily task of duties.

### Attitudes

In the study [20], there were no statistically significant improvements in attitudes towards oral health care following the intervention, compared to the situation before the intervention. The study [18] reported that the majority of healthcare professionals (77%) were comfortable performing oral procedures and adequately trained to provide oral health care instructions (72%) and perform oral care procedures (84%). Additionally, 70% of participants were very confident in examining for the presence of oral pain and providing oral hygiene instructions. However, less than half of the participants were very confident in their ability to examine the health of teeth and gums for complications of trismus, dysphagia, and xerostomia. A study [19] reported that 56.9% of nurses feeling comfortable while examining a patient’s oral cavity.

## Discussion

The findings from the reviewed studies offer valuable insights into the knowledge, practices, and attitudes of healthcare providers, particularly nurses, concerning oral care for oncology patients undergoing cancer treatment or hematopoietic stem cell transplantation (HSCT). These insights have significant implications for improving oral healthcare within this specific patient population.

The studies reviewed consistently indicate a lack of knowledge and skills among healthcare providers, specifically nurses, when it comes to oral care for cancer patients undergoing treatment. This knowledge gap is evident in their ability to make accurate diagnoses, administer appropriate treatments and hygiene protocols, and address challenges related to the diagnosis and treatment of xerostomia. Furthermore, the studies highlight variations in practice among different healthcare groups, underscoring the need for education and training in oral care.

These findings align with an Australian study that focused on cardiovascular disease (CVD), where the majority of participants (*n* = 24) reported limited or no knowledge about the link between oral health and CVD. Similarly, other non-dental healthcare professionals, such as antenatal care providers, have been reported to lack oral health knowledge [21]. In terms of clinical practice, the study found that patients with CVD received no regular oral health education, and healthcare providers received no oral health training. Positive advancements have been observed in the United States, where oral health has been incorporated into nursing courses as part of a nationwide strategy aimed at enhancing the quality of oral healthcare and addressing disparities [22]. Additionally, an Australian university successfully integrated an oral health module into an undergraduate midwifery course [23]. These efforts signify important steps forward in improving oral care education and training within the healthcare profession. The reviewed studies highlight the need for improved knowledge and practices in oral care among healthcare providers, particularly nurses, who work with oncology patients undergoing cancer treatment or HSCT.

The studies in this review revealed varying levels of knowledge among nurses regarding oral diagnosis, treatment, disease etiologies, and hygiene protocols. While nurses demonstrated proficiency in giving proper differential diagnoses of mucositis and knowledge of proper treatment for fungal infections, they faced challenges in diagnosing and treating xerostomia. Furthermore, nurses exhibited less than adequate knowledge on how to provide oral care for children undergoing cancer treatment and HSCT. These findings highlight the need for targeted educational interventions to enhance nurses’ knowledge and understanding of oral care practices in oncology settings.

The studies emphasized the importance of improving oral healthcare practices among healthcare providers. Referral to dental services emerged as a crucial avenue for children to receive comprehensive oral care during cancer treatment or prior to HSCT. However, it was observed that only a minority of healthcare providers made such referrals, indicating a gap in coordination between healthcare and dental professionals. Additionally, there were deficiencies in conducting thorough examinations for complications such as xerostomia and trismus. These findings underscore the necessity of standardized protocols, comprehensive assessments, and early intervention strategies to enhance oral care practices in oncology settings. A study among nurses in Turkey highlighted that 53.5% of nurses reported their clinics did not have a standard oral care protocol, 41.6% reported having an oral care protocol and implementing it, and 4.9% reported having an oral care protocol but not implementing it. The statistical evaluation revealed a statistically significant difference between the hospitals in terms of performing oral care, conducting oral assessments regularly, and implementing oral care protocols [24].

Regarding attitudes towards oral care, a discrepancy exists between nurses’ confidence in performing oral care procedures and their ability to provide oral hygiene instructions. This suggests that while they may possess the technical skills, they may lack the necessary knowledge to educate patients about oral care. Moreover, the studies highlight the need for improved oral healthcare practices and referrals for pediatric oncology patients undergoing cancer treatment or HSCT. The studies presented mixed attitudes among healthcare providers regarding oral healthcare. While a majority of participants felt comfortable performing oral procedures and were adequately trained to provide oral health care instructions and perform oral care procedures, there were areas of lower confidence. Participants expressed less confidence in examining the health of teeth and gums for complications such as trismus, dysphagia, and xerostomia. These findings highlight the need for targeted training programs to address specific areas of lower confidence and enhance overall attitudes towards comprehensive oral care. This is consistent with a recent Saudi study that found significant issues regarding the current practice of oral care for hospitalized patients in Saudi hospitals. The study revealed that not all patients underwent oral health assessment, indicating a gap in providing comprehensive care. Additionally, some hospitals lacked clear published policies regarding oral care for hospitalized patients, leading to inconsistencies in practice. The documentation of oral care procedures was also problematic, and there were barriers hindering the provision of adequate oral care to patients. The study emphasized the need to enhance the training and continuous education of nurses in Saudi Arabia concerning oral care for hospitalized patients. The majority of participating nurses expressed a deficiency in their training on oral assessment and the provision of oral care [11]. Another Australian study also highlighted the significance of oral health among nurses, acknowledging that patient behaviors can impact their ability to carry out oral care tasks effectively. To address this issue, it is recommended that educational institutions and hospitals collaborate to develop a formal oral health procedure and training package specifically tailored for acute geriatric care wards [25].

Furthermore, a recent study conducted by [26] discovered significant associations between oral hygiene practices and various factors. These factors included the availability of working wards, the level of qualification, the presence of oral health care guidelines, access to specific resources, and previous training in oral care. The study emphasized the importance of providing oral hygiene practices for hospitalized stroke patients. However, it also revealed the detrimental effects of a lack of oral health care guidelines, insufficient support from dental professionals, inadequate resources, inadequate training, and the absence of assistance in daily oral care for patients.

Comparing these results with global literature, it is evident that the lack of knowledge and skills among healthcare providers regarding oral care is not unique to a specific region or country. Similar studies conducted in other countries, such as the USA, Australia, Turkey, and Saudi Arabia [26, 25, 27], have found comparable results. However, some studies have shown that interventions, such as education and training programs, can improve the knowledge and skills of oncology healthcare providers and lead to improved oral care practices. Therefore, it is important to implement such interventions to address the gaps in knowledge and skills among healthcare providers regarding oral care.

## Conclusion

In conclusion, the included studies showed a diversity in practice among groups, a lack of oral care knowledge, and the pertinent need for education. Therefore, more attention should be given to developing standardized protocols or clinical practice guidelines for oral care for postoperative patients with oral cancer to equip nurses with the required skills to deliver quality oral care. Additionally, continuing education and training programs in oral care for nurses should be developed to improve their knowledge and skills in this area.

## Strength and limitations

The study conducted a systematic review of primary quantitative and qualitative studies, thoroughly examining nurses’ knowledge, attitudes, and practices regarding oral healthcare. To ensure a comprehensive analysis, multiple reputable healthcare databases, including CINAHL, Cochrane, Medline, PubMed, ScienceDirect, Scopus, and Google Scholar, were extensively searched. The use of standardized tools such as the JBI System for Unified Management, Assessment, and Review of Information (JBI SUMARI) and the JBI extraction tool ensured consistency and reliability throughout the data collection and extraction processes.

However, it is important to acknowledge certain limitations of the study. The reported results predominantly focus on pediatric patients. It is important to note that these two groups, pediatric and adult patients, are distinct, which represents a notable limitation of this Systematic review. Moreover, by excluding non-peer-reviewed and gray literature, the study may have missed out on valuable unpublished research, leading to publication bias. Additionally, the study’s time frame was limited to articles published between 2000 and 2023, which may have excluded older studies that could have provided valuable insights on the topic.

Despite these limitations, the systematic review provides a comprehensive overview of the existing evidence on nurses’ knowledge, attitudes, and practices regarding oral healthcare, offering valuable insights into this important aspect of healthcare delivery.

## Implications

The finding shows diversity in practice among groups, lack of oral care knowledge, and the pertinent need for education. There is a clear need for targeted education and training programs for healthcare providers, especially nurses, working with oncology patients undergoing cancer treatment and palliative patients. These programs should focus on enhancing knowledge and understanding of oral care practices, including accurate diagnosis, appropriate treatment, and proper hygiene protocols. Implementation of comprehensive educational interventions can bridge the knowledge gap and improve oral care practices (grade A).

It is essential to establish standardized oral care protocols in healthcare settings to ensure consistent and evidence-based practices. The development and implementation of these protocols should involve collaboration between healthcare and dental professionals. Standardized protocols can guide healthcare providers in conducting thorough examinations, addressing complications, and providing appropriate referrals to dental services (grade A).

There is a need to improve oral health assessment practices and documentation in healthcare settings. Healthcare providers should be trained to conduct comprehensive assessments, including examinations for complications related to oral health. Clear and standardized documentation of oral care procedures is essential for maintaining continuity of care and facilitating communication between healthcare providers (grade B).

### Supplementary information

Below is the link to the electronic supplementary material.

Supplementary file1 (docx 16.4 KB)

## Data Availability

Data sharing is not applicable to this article as no datasets were generated or analyzed during the current study.
