# Supplementary material for: The intersection of oncology and oral health: exploring nurses’ insights and practices — a systematic review
Source: Support Care Cancer. 2024 Jan 30;32(2):138. doi: 10.1007/s00520-024-08317-5 (PMC10827822; doi:10.1007/s00520-024-08317-5)
Supplement: Supplementary file 1 — Supplementary file1 (docx 16.4 KB) [file 520_2024_8317_MOESM1_ESM.docx]

**Appendix A: Excluded Studies**

| **S.NO** | **Excluded Studies** | **Reasons of exclusion** |
| --- | --- | --- |
|  | Xiaoxian M, Duncan RP, Porter CK, Qing L, Tomar SL. Florida nurse practitioners’ attitudes and practices regarding oral cancer prevention and early detection. Journal of the American Academy of Nurse Practitioners. 2007;19(12):668-75. Available from: https://doi.org/10.1111/j.1745-7599.2007.00274.x | Focused on oral cancer, not the oral care of cancer survivors |
|  | Wong G, Koo T, Fethney J, Chen R. Assessing oral health literacy of university nursing students: A cross-sectional exploratory study. Nurse Education in Practice. 2021;53:N.PAG-N.PAG. Available from: https://doi.org/10.1016/j.nepr.2021.103066 | Focused on the nursing students' knowledge |
|  | Walker K, Jackson R, Edwards PC, Vadaparampil ST. HPV and Oral Cancer: The need to integrate oral health practices into nursing education. Clinical Journal of Oncology Nursing. 2018;22(6):E166-E73. Available from: https://doi.org/10.1188/18.CJON.E166-E173 |  |
|  | Ahmad MS, Abuzar MA, Razak IA, Rahman SA, Borromeo GL. Perceptions of oral health education and practice among nursing students in Malaysia and Australia. International Journal of Dental Hygiene. 2021;19(2):215-22. Available from: https://doi.org/10.1111/idh.12488 |  |
|  | Clemmens D, Rodriguez K, Leef B. Knowledge, attitudes, and practices of baccalaureate nursing students regarding oral health assessment. The Journal of nursing education. 2012;51(9):532-5. Available from: https://doi.org/10.3928/01484834-20120820-01 |  |
|  | Honnor A, Law A. Mouth care in cancer nursing: using an audit to change practice. British Journal of Nursing. 2002;11(16):1087-96. Available from: https://doi.org/ 10.12968/bjon.2002.11.16.10550 | Mixed methodology study |
|  | Ruegg TA, Morse JM, Yechieli RL. Nurse-Delivered Telephone Intervention to Reduce Oral Mucositis and Prevent Dehydration. Oncology Nursing Forum. 2021;48(2):242-56. Available from: https://doi.org/10.1188/21.ONF.242-256 |  |
|  | Li HW, Huang CH, Chou C, Wang TF. Knowledge, attitudes, practice and related factors of oral cancer prevention among public health nurses in Taiwan. European Journal of Cancer Care. 2020;29(5):1-9. Available from: https://doi.org/10.1111/ecc.13262 | Only public health nurses’ oral cancer knowledge, attitudes and no oral health care management or interventions |
|  | Foulkes M. Oral cancer: risk factors, treatment and nursing care. Nursing standard (Royal College of Nursing (Great Britain) : 1987). 2013;28(8):49-57. Available from: https://doi.org/10.7748/ns2013.10.28.8.49.e7593 | Not study on nurse's knowledge, attitudes, and practices towards oral healthcare |
|  | Cadet T, Maramaldi P, Burke SL, LeCloux M, White E, Kalenderian E, et al. Oral health and cancer screening in long-term care nursing facilities: Motivation and opportunity as intervention targets. Gerodontology. 2018;35(4):407-16. Available from: https://doi.org/10.1111/ger.12365 |  |
|  | Bordonaro S, Raiti F, Di Mari A, Lopiano C, Romano F, Pumo V, et al. Active home-based cancer treatment. Journal of multidisciplinary healthcare. 2012;5:137-43. Available from: https://doi.org/10.2147/JMDH.S31494 |  |
|  | Wee WMY, Ang E, Ng PI. Oral hygiene of patients with cancer in an acute oncology ward: a best practice project. International Journal of Evidence-Based Healthcare. 2013;11(3):194-201. Available from: https://doi.org/10.1111/1744-1609.12028 | Small sample size in the study and exposure not measured in a valid and reliable way |
|  | Ohrn KE, Wahlin YB, Sjödén PO. Oral care in cancer nursing. European journal of cancer care. 2000;9(1):22-9. Available from: https://doi.org/10.1046/j.1365-2354.2000.00185.x | Statistical analysis was not appropriate |
